# Anterior Urethral Valves: Not Such a Benign Condition…

**DOI:** 10.3389/fped.2013.00035

**Published:** 2013-11-08

**Authors:** Omar Cruz-Diaz, Anahi Salomon, Eran Rosenberg, Juan Manuel Moldes, Francisco de Badiola, Andrew Scott Labbie, Rafael Gosalbez, Miguel Alfredo Castellan

**Affiliations:** ^1^Division of Pediatric Urology, Miami Children’s Hospital, Miami, FL, USA; ^2^Joe Di Maggio Children’s Hospital, Hollywood, FL, USA; ^3^Servicio de Urologia Pediatrica, Hospital Italiano de Buenos Aires, Buenos Aires, Argentina

**Keywords:** anterior urethral valves, urethral diverticulum, end-stage renal disease, hydronephrosis, urinary tract obstruction

## Abstract

**Purpose:** Anterior urethral valves (AUVs) is an unusual cause of congenital obstruction of the male urethra, being 15–30 times less common than posterior urethral valves (PUVs). It has been suggested that patients with congenital anterior urethral obstruction have a better prognosis than those with PUV, with less hydronephrosis, and a lower incidence of chronic renal insufficiency (5 vs. 30%). The long-term prognosis of AUVs is not clear in the literature. In this report we describe our experience and long-term follow up of patients with anterior urethral valve.

**Materials and Methods:** We retrospectively identified 13 patients who presented with the diagnosis of AUVs in our institutions between January 1994 and June 2012. Two patients were excluded: one patient had no follow up after intervention; the other had a follow up <1 year. From the 11 patients included, we evaluated the gestational age, prenatal and postnatal ultrasound findings, voiding cystourethrogram findings, age upon valve ablation, micturition pattern, creatinine, and clinical follow up.

**Results:** Between 1994 and 2012 we evaluated 150 patients with the diagnosis of urethral valves. Of this group, 11 patients (7.3%) had AUVs and an adequate follow up. Mean follow up is 6.3 years (2.5–12 years). Five (45.4%) patients had prenatal diagnosis of AUV. The most common prenatal ultrasonographic finding was bilateral hydronephrosis and distended bladder. One patient showed a large perineal cystic mass, which was confirmed to be a dilated anterior urethra. The mean gestational age was 37.6 weeks (27–40 WGA). Postnatally, 90% had trabeculated bladder, 80% hydronephrosis, and 40% renal dysplasia. The most common clinical presentation was urinary tract infection in five patients (45.4%), followed by weak urinary stream found in four patients (36.3%). The age at initial surgical intervention ranged between 7 days and 13 years. Seven (63.6%) patients had primary transurethral valve resection or laser ablation and three patients (27.2%) had primary vesicostomies. One boy (9.1%) had penile urethrostomy with excision of urethral diverticulum. Two (18.2%) patients developed end-stage renal disease.

**Conclusion:** Anterior urethral valve is a rare congenital entity affecting the genitourinary system in males. Early urinary tract obstruction resulted in end-stage renal disease in 18% of our patient population. In our series, the complication rate and the evolution to renal failure are high and similar to patients with PUV. In patients with AUVs we recommend long-term follow up and close evaluation of patient’s bladder and renal function.

## Introduction

Anterior urethral valves (AUVs) are the most common congenital obstructive disorder affecting the male anterior urethra (1). Nonetheless, it is still quite rare and occur 15–30 times less frequently than posterior urethral valves (PUVs) (2). An anterior urethral valve is a posteriorly directed semilunar fold arising from the floor of the anterior urethra and causing urethral obstruction during micturition (3, 4).

Anterior urethral valves can be located anywhere distal to the membranous urethra, 40% are found in the bulbar urethra, 30% at the penoscrotal junction, and 30% in the penile urethra (5, 6). Possible causes of anterior urethral valve have been considered including imbalance of tissue growth during urethral development, incomplete hypospadias, or an abortive process during urethral duplication and a faulty union between glandular and urethral tissue during embryonal development.

**Figure 1:**
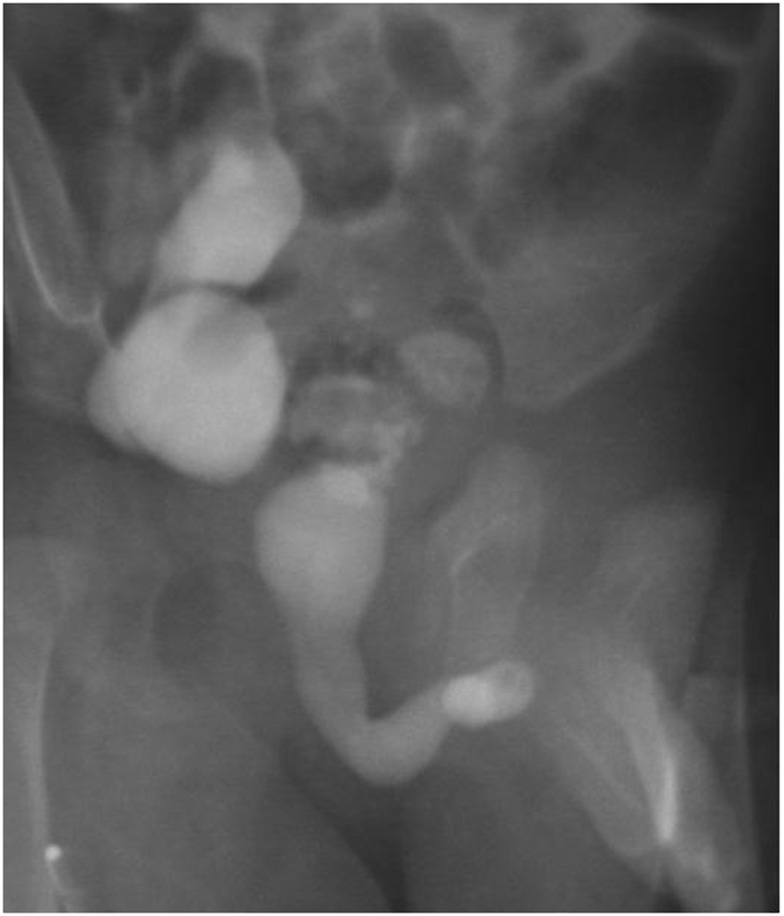
**Voiding cystourethrogram (VCUG) showing thickened bladder neck and dilated urethra proximal to the anterior urethral valve**.

Patients with AUVs have a highly variable clinical presentation depending on age and degree of urinary obstruction. In some cases, symptoms may be very subtle; others may develop urinary incontinence, urinary retention, weak urinary stream, post-micturitional dribbling, bulging on the ventral penis, urinary tract infection, and urosepsis (7–9). Because of this variability, diagnosis requires a high degree of suspicion. The diagnostic study of choice is a voiding cystourethrogram (VCUG) that may show a trabeculated bladder, bladder diverticulum, urethral diverticulum, and/or a dilated urethra proximal to the valve (Figure 1). On ultrasonography patients could demonstrate bladder distension and bilateral hydroureteronephrosis.

Anterior urethral valve is a rare condition and, it is not clear in the literature if patients with AUV have a better prognosis than patients with PUV (10). The purpose of this study is to discuss our experience on patients with anterior urethral valve with a longer term follow up.

## Materials and Methods

This study was approved by the Institutional Review Board Committee of our institution.

We retrospectively evaluated patients who presented with anterior urethral valve at Miami Children’s Hospital, Joe DiMaggio Children’s Hospital in Miami, FL, USA, and Hospital Italiano in Buenos Aires, Argentina. Between 1994 and 2011 we identified 150 patients with urethral valves, and from this group 13 patients (8.6%) had AUVs. Two patients were excluded because they did not have an adequate follow up after intervention. From the 11 patients included, we evaluated the gestational age, prenatal and postnatal ultrasound findings, VCUG findings, age upon valve ablation, micturition pattern, creatinine, and clinical follow up.

## Results

**Figure 2:**
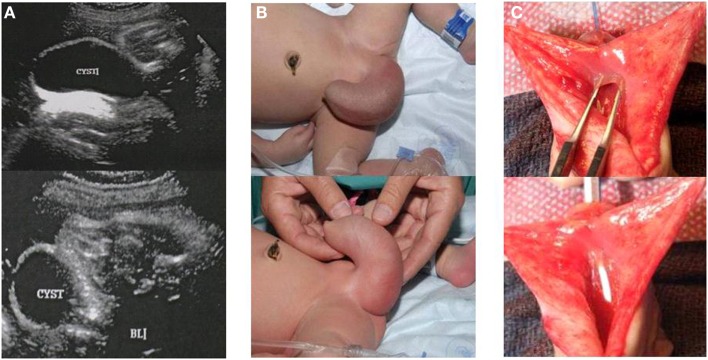
**Male patient with anterior urethral diverticulum**. **(A)** Prenatal ultrasound showing large cystic mass in the genital area. **(B)** Enlarged urethral bulging mass, megalourethra. **(C)** Picture depicting anterior urethral valve during open urethroplasty.

Eleven (11/13) patients (7.3%) had adequate follow up and were included in this study. Mean follow up was 6.3 years (2.5–12 years). Five patients (45.4%) had prenatal diagnosis of AUV. The most common prenatal ultrasonographic finding was bilateral hydronephrosis and distended bladder. One patient showed a large perineal cystic mass of about 5 cm in diameter and after birth it was confirmed to be a megalourethra (Figure 2). Mean gestational age was 37.6 weeks (27–40 WGA). Seven patients (63.6%) were born at term and four patients (36.3%) were premature.

Eight patients (72.7%) had a neonatal presentation; the other three patients were diagnosed at 2, 8, and 13 years of age. The most common clinical presentation was urinary tract infection in five patients (45.4%), followed by weak urinary stream found in four patients (36.3%). Other clinical manifestations included lower urinary tract obstruction, palpable distended bladder, diurnal/nocturnal urinary incontinence, and renal insufficiency. As described above, one patient was diagnosed at birth because of megalourethra.

Table 1 describes in detail pre-operative sonographic findings. Ten patients (90.9%) had pre-operative ultrasonographic imaging available, 9 patients (90%) had trabeculated bladder, 8 (80%) hydronephrosis (6 patients bilateral, 2 patients unilateral), and 4 (40%) renal dysplasia (2 patients bilateral, 2 patients unilateral).

**Table 1:** **Renal and bladder ultrasound pre-operative findings**.

Patient	Hydronephrosis	Bladder trabeculation	Renal dysplasia	Retrovesical ureter	Paraurethral diverticulum
1	Bilateral	+	−	−	−
2	Bilateral	+	−	Left	−
3	Bilateral	+	Left	−	−
4	Right	+	−	−	−
5	Bilateral	+	Bilateral	−	Right
6	Bilateral	+	Bilateral	Bilateral	−
7	Bilateral	+	−	−	−
8	−	+	−	−	−
9	*	*	*	*	*
10	Right	+	Left	−	−
11	−	+	−	−	−

−, None; +, present; *, information not available.

Ten patients out of 11 (90.9%) had pre-operative VCUG available for evaluation (Table 2). Seven patients (70%) had thickened bladder with trabeculations, four (40%) vesicoureteral reflux, and one a patent urachus. One case did not have a VCUG because a cutaneous urethrostomy was done in the neonatal period.

**Table 2:** **Pre- and post-operative VCUG findings**.

Patient	Pre-operative VCUG				Post-operative VCUG			
	Radiologic diagnosis	Bladder trabeculations	VUR	PUD	Radiologic diagnosis	Bladder trabeculations	VUR	PUD
1	AUV	+	−	−	Normal	+	−	Right
2	Anterior urethra dilation	+	Right	−	Mild urethral dilation	+	−	−
3	AUV	−	B/L (I/IV)	−	Normal	+	Left (IV)	−
4	Anterior/bulbar urethral dilation	+	−	Right	Normal	+	−	B/L
5	Posterior urethral stricture	+	−	B/L	Normal	−	Right (V)	−
6	AUV and patent urachus	+	−	−	Moderate urethral dilation	−	−	−
7	Urethral dilation	−	Right (V)	B/L	Normal	−	−	−
8	Anterior urethral dilation	+	B/L (III/IV)	−	Normal	+	B/L (I/III)	−
9	*	*	*	*	*	*	*	*
10	AUV	+	−	−	Urethral dilation improvement	−	−	−
11	High PVR	−	−	−	Normal	−	−	−

AUV, anterior urethral valve; VUR, vesicoureteral reflux; PUD, paraureteral diverticulum; B/L, bilateral; *, information not available.

Age at initial surgical intervention ranged between 7 days and 13 years (mean age: 2.2 years). Seven patients (63.6%) had primary transurethral resection or laser ablation of the anterior urethral valve. Three patients (27.3%) had primary vesicostomy, done mainly because of prematurity, low birth weight, and increased creatinine levels. One boy (9.1%) had a primary cutaneous urethrostomy for correction of dilated anterior urethra (Picture 2). After initial valve ablation, 4/7 (57%) patients did not require any additional surgical procedure. One patient (adolescent) developed a urethral stricture about 12 months after valve ablation, requiring a one-stage urethroplasty. Since surgery, patient has been voiding spontaneously without further complications. In two patients a bilateral vesicoureteral reimplantation was done because of vesicoureteral reflux after initial valve ablation. Currently both are doing well, with a normal creatinine and post-operative VCUG without reflux. Both patients had open ureteral reimplantation due to concomitant bilateral bladder diverticulectomy in the first one and bilateral ureteral tailoring due to bilateral megaureters in the second patient.

In three patients a primary vesicostomy was done in the neonatal period. In one patient vesicostomy was closed at 7 months of age with resection of the valve. Patient did very well and did not require any additional surgical procedure. He is 5 years of age now and has symptoms of urgency and frequency but with a normal renal function. Another patient at 16 months of age vesicostomy was closed and the valve ablated but, at 27 months of age patient’s vesicostomy needed to be redone because of recurrent urinary tract infections and renal function deterioration. Currently patient is on waiting list for renal transplant. The third patient with a vesicostomy had clinical appearance resembling prune belly syndrome, with history of renal failure and bilateral renal dysplasia. A bilateral ureteral reimplantation was done at the age of 2 years. Also an appendicovesicostomy was done for intermittent catheterization due to elevated residual. At the age of 6 years, patient received a cadaveric renal transplant.

In terms of bladder function assessment, the majority of our patients were potty trained, with adequate flow pattern and minimal post-void residuals. These patients were followed clinically and with serial renal and bladder ultrasounds. On the other hand, one patient needed the cutaneous vesicostomy to be re-opened due to renal function deterioration; currently he is in waiting list for renal transplant and micturition and urodynamic evaluation is not possible at this point. Other patient had history of prune belly, megaureters, rejected cadaveric kidney transplant and currently is on clean intermittent catheterization. This patient had an urodynamic evaluation showing small bladder capacity with adequate compliance, stable detrusor, and sensation during bladder filling. Patient voided with poor flow with an average of 4 ml/s, a maximum flow of 11 ml/s with a significant abdominal voiding pattern and complete bladder emptying.

Three patients (27.3%) showed creatinine elevation before primary treatment. One patient had creatinine normalization after ablation, whereas the other two patients developed chronic renal insufficiency. Nine patients (90.9%) currently void spontaneously, one patient is on clean intermittent catheterization, and the other patient still has an open vesicostomy due to renal insufficiency and he is on the waiting list for transplant (Table 3). One patient develops symptoms of urinary urgency and frequency, and requires treatment with oral anticholinergic therapy.

**Table 3:** **Pre- and post-operative renal function, voiding pattern, urinary incontinence, and dialysis requirement**.

Patient	Pre-operative cretinine	Post-operative creatinine	Voiding	Incontience	Dialysis
1	Normal	Normal	Spontaneous	−	−
2	Elevated	Normal	Spontaneous	−	−
3	Elevated	Elevated	Vesicostomy	−	+
4	Normal	Normal	Spontaneous	−	−
5	Normal	Normal	Spontaneous	−	−
6	Elevated	Elevated	CIC	−	+
7	Normal	Normal	Spontaneous	−	−
8	Normal	Normal	Spontaneous	−	−
9	Normal	Normal	Spontaneous	Frequency/urgency	−
10	Normal	Normal	Spontaneous	−	−
11	Normal	Normal	Spontaneous	−	−

CIC, clean intermittent catheterization.

## Discussion

Congenital anterior urethral obstruction is a disorder that is less frequent than PUV. In general terms, in cases where prenatal diagnosis is not available a high degree of suspicion is required for diagnosis.

Voiding cystourethrogram remains the gold standard imaging modality for the diagnosis of urethral anomalies (4, 11). It is imperative to obtain and evaluate the voiding phase to avoid missing the diagnosis of AUV. Retrograde urethrography has not been evaluated and cystoscopic evaluation of neonatal urethras may be quite challenging considering the small urethral meatus and difficult visualization of the distal penile urethra (3).

Some authors distinguish between anterior urethral valve from anterior urethral diverticulum based on anatomical, radiological, and embryological differences (8). The diverticulum is associated with a distal lip-like tissue which may be confused with a valve. It has been suggested that the embryological origin of anterior urethral diverticula could be a flaw in the union between glandular and penile urethral segments or due to incomplete spongy tissue formation (7). On the other hand, patients with AUV have normal corpus spongiosum development (12). McLellan et al. suggested that anterior urethral diverticula may result from rupture of dilated bulbourethral glands (13). In our series, one patient was diagnosed with anterior urethral diverticulum and required resection of diverticulum and staged urethroplasty.

Radiologically, in patients with AUV the proximal part of the AUV forms an obtuse angle with the ventral floor whereas the proximal lip of a diverticulum forms an acute angle with the ventral floor of the urethra (11). During voiding, the urethral diverticulum is distended, compressing the distal lip and obstructing the urinary flow (3).

Patients with AUV that are diagnosed prenatally, usually present with bilateral hydronephrosis and in severe cases with megaureters and/or megacystis. One patient in our series with prenatal diagnosis of AUVs developed megacystis and pseudo Prune Belly. The postnatal ultrasound revealed bilateral renal dysplasia and required vesicostomy during the neonatal period. Later on, patient had bilateral ureteral reimplantation, closure of vesicostomy, and a continent catheterizable stoma. Renal function deteriorated and eventually patient received a cadaveric kidney transplant.

Initial management of patient with the suspicion or diagnosis of anterior urethral valve is placement of indwelling urethral catheter to allow decompression and drainage of the genitourinary system. This may help reducing the risk of urosepsis and further renal function deterioration and may promote the stabilization of any electrolytes imbalance. Surgical correction is the definitive management of an AUV.

In the past, surgical options to correct AUVs included open urethrotomy and excision of the valve and segmental uretherectomy of the valve-bearing area along with a primary end-to-end anastomosis (14, 15).

Transurethral valve ablation, either with laser, electrocautery, or cold-cut, has been described as a very effective modality, considering the advanced endoscopic instrumentation currently available. In patients with very low birth weight, where endoscopic instrumentation may be challenging, a temporary cutaneous vesicostomy may be used to prevent possible complications such as urethral stricture and inadequate valve ablation due to very small urethral size (16). Cases with anterior urethral diverticula can be treated in one or multiple stages, with diverticulectomy and urethroplasty, requiring in some instances cutaneous urethrostomy proximal to the lesion, which has the advantage of avoiding bladder function deterioration as compared with vesicostomy (12, 17).

Among surgical complications described in the literature are scrotal urinoma/hematoma, urethral strictures, and urethrocutaneous fistulae (18). In our series, one patient developed a urethral stricture 1 year after endoscopic resection of AUVs, requiring a single stage urethroplasty. Currently this patient is asymptomatic and has a normal voiding pattern. None of our patients developed urethrocutaneous fistulae. Between 75 and 80% of patients with anterior urethral valve present with some degree of bladder dysfunction, even after adequate valve ablation is done (19). Kajiwara et al. evaluated urodynamic characteristics in patients with AUV which included bladder instability, overactive bladder, and poor bladder compliance and capacity (20).

In terms of prognostic factors, Routhe et al. noted that patients with AUVs with high pre-operative creatinine levels associated with vesicoureteral reflux and urinary tract infections had a 25-fold increase in poor renal outcomes (19). In our series, three patients (28%) had high pre-operative creatinine levels. From these three patients only one patient (33%) had normalized creatinine values after successful valve ablation. Among the patients with vesicoureteral reflux, only those with low-grade VUR (Grade I–II) achieved improvement or resolution of reflux, while patients with high-grade VUR (Grade III–V) persisted with reflux and required ureteral reimplantation.

Routh et al. described that from a population of 132 patients, 31 patients (22%) did not have a normal renal function after anterior valve treatment (19). Of these 31 children, 17 (12%) had stable azotemia, 5 (4%) progressed to end-stage renal disease requiring dialysis or transplantation, and 9 (6%) died. In our series, two patients (18%) developed end-stage renal disease; one patient received a cadaveric renal transplant; and the other patient required his vesicostomy to be re-opened and is on the waiting list for renal transplant as well. Our results showed a higher incidence of end-stage renal disease in patients with AUVs.

Limitations of this study include its retrospective nature as well as the limited size of our population. Considering the rarity of AUVs, this size is comparable to others series previously described in the literature.

## Conclusion

Anterior urethral valve is a rare congenital disorder affecting the genitourinary system in male patients. A high degree of suspicion is required for diagnosis considering the subtle symptomatology of AUVs. In this series, early urinary tract obstruction resulted in end-stage renal disease in 18% of our patients. There may exist a false perception that anterior urethral valve is a benign condition possibly related to the clinical presentation described above. The complication rate and the evolution to renal failure are high and this group of patients needs to be followed-up closely. In patients with AUVs we recommend a long-term follow up with special attention to their bladder and renal function.

## Conflict of Interest Statement

The authors declare that the research was conducted in the absence of any commercial or financial relationships that could be construed as a potential conflict of interest.
